# Glucagon‐Like Peptide‐1 Agonist vs. Placebo and Pulmonary Decline After Open‐Heart Surgery: A Substudy of the GLORIOUS Randomised Clinical Trial

**DOI:** 10.1111/aas.70282

**Published:** 2026-06-19

**Authors:** Astrid Duus Mikkelsen, Sebastian Wiberg, Hans Henrik Lawaetz Schultz, Peter Hasse Møller‐Sørensen, Dan Høfsten, Jens Christian Nilsson, Christian Holdflod Møller, Lars Køber, Christian Hassager, Jesper Kjærgaard

**Affiliations:** ^1^ Department of Cardiology Copenhagen University Hospital, Rigshospitalet Copenhagen Denmark; ^2^ Department of Cardiothoracic Anaesthesiology and Intensive Care Copenhagen University Hospital Copenhagen Denmark; ^3^ Department of Clinical Medicine University of Copenhagen Copenhagen Denmark; ^4^ Department of Cardiology, Section for Heart‐and Lung Transplant Copenhagen University Hospital Copenhagen Denmark; ^5^ Department of Cardiac Surgery Copenhagen University Hospital Copenhagen Denmark

**Keywords:** cardiopulmonary bypass, DLCO, FEV_1_/FVC, glucagon‐like peptide‐1 receptor agonist, open‐heart surgery, postoperative pulmonary decline, pulmonary protection

## Abstract

**Background:**

Postoperative pulmonary decline is an established complication of open‐heart surgery extending beyond the immediate postoperative phase. Inflammation‐mediated lung damage and ischaemia‐reperfusion injury secondary to extracorporeal circulation is a proposed pathophysiological driver. GLP‐1 receptor agonists (GLP‐1RA) have emerged as promising protective agents in this setting.

**Aim:**

Investigate whether infusion of the GLP‐1RA, exenatide during cardiopulmonary bypass and weaning thereof, can mitigate the decline in diffusing capacity and ventilatory performance 3 months postoperative, compared to placebo.

**Methods:**

In this predefined explorative substudy of the randomised, clinical GLORIOUS trial, 878 adult patients undergoing non‐emergent coronary artery bypass grafting (CABG) and/or surgical aortic valve replacement (SAVR) were randomised to a continuous infusion of the GLP‐1RA, exenatide or placebo during cardiopulmonary bypass, extending into the early postoperative period. Diffusing capacity of the lung for carbon monoxide (DLCO) and ventilatory performance (FEV_1_/FVC) were measured preoperatively and 3 months postoperatively.

**Results:**

Median DLCO (% predicted corrected) declined from 80% preoperative to 72% 3 months postoperative, corresponding to a −7.7 percentage point (pp) difference (95% CI 6.2 to 9.1; *p* < 0.001). FEV_1_/FVC declined from 0.75 preoperative to 0.73 postoperative, corresponding to a −1.6 difference (95% CI 1.0 to 2.1; *p* < 0.001). However, there were no significant differences in decline between the exenatide and placebo groups (all *p* > 0.3). Findings were consistent across subgroup analyses.

**Conclusion:**

While both diffusing capacity and ventilatory performance exhibited a mild‐to‐moderate decline 3 months after open‐heart surgery, the GLP‐1RA exenatide did not mitigate this decline compared with placebo.

**Editorial Comment:**

Pulmonary dysfunction is one of the most common complications to open‐heart surgery. The present study confirms a decline in diffusing capacity of the lung for carbon monoxide (DLCO) and in ventilatory performance measured as FEV_1_/FVC at 3 months postoperatively compared to preoperative measurements. Infusion of GLP‐1 receptor antagonist during cardiopulmonary bypass and weaning did not mitigate the pulmonary decline compared to placebo.

## 1 Introduction

Pulmonary dysfunction is a known complication of open‐heart surgery well beyond the early postoperative period [1, 2, 3, 4]. Despite advances in perioperative management and surgical techniques, recent studies report a decline in pulmonary diffusing capacity and ventilatory function of up to 13% 4 months after surgery [1, 2]. The massive immune activation triggered by extracorporeal circulation of native blood during cardiopulmonary bypass (CPB) is a proposed pathophysiological mechanism [5, 6, 7]. The pulmonary reperfusion phase during weaning from CPB is considered particularly vulnerable to inflammation‐mediated pulmonary tissue damage [7, 8]. However, the topic remains sparsely investigated, and data from randomised clinical trials are lacking.

Glucagon‐like peptide‐1 receptor agonists (GLP‐1RA) have in recent years demonstrated promising results in organ protection beyond their insulinotropic properties [9, 10, 11, 12]. Preclinical studies have shown anti‐inflammatory actions in acute lung injury and mitigation of pulmonary fibrosis [13, 14]. Clinical studies have demonstrated that GLP‐1RA can improve forced vital capacity (FVC) and diffusing capacity of the lungs for carbon monoxide (DLCO) in adult patients with chronic lung disease [15, 16]. The GLP‐1RA exenatide has been shown to reduce reperfusion injury in ST‐segment elevation myocardial infarction (STEMI) patients [9]. Human lung tissue possesses a high density of GLP‐1 receptors, making this class of drugs an interesting target for pulmonary protection in CPB‐assisted open‐heart surgery.

With this study, we aimed to explore whether a GLP‐1RA could mitigate postoperative pulmonary decline in patients undergoing non‐emergent CPB‐assisted open‐heart surgery. We hypothesized that infusion with the GLP‐1RA, exenatide during CPB and early postoperative phase, reduced the decline in diffusing capacity and ventilatory performance compared with placebo at 3 months postoperative.

## 2 Methods

### 2.1 Study Design and Population

This is a predefined, exploratory substudy of the GLP‐1 agOnist and Restrictive versus lIberal FiO_2_ in patients Undergoing open‐heart Surgery (GLORIOUS) trial—an investigator‐initiated, single‐centre, randomised, placebo‐controlled, two‐by‐two factorial design, clinical trial (ClinicalTrials.gov identifier NCT02673931) [17]. The GLORIOUS trial was approved by the Regional Ethics Committee of the Capital Region of Denmark (H‐15010562), the Danish Medicines Agency (EudraCT 2015–003050‐41), and the Danish Data Protection Agency (ID RH‐2016‐23).

The GLORIOUS trial investigated a perioperative GLP‐1RA vs. placebo, and restrictive fraction of inspired oxygen (FiO_2_ 50%) versus liberal (FiO_2_ 100%) perioperative oxygenation strategy in adult patients undergoing CPB‐assisted open‐heart surgery. Inclusion criteria comprised age above 18 years, non‐emergent coronary artery bypass‐grafting (CABG) and/or surgical aortic valve repair (SAVR), irrespective of concomitant aorta and/or heart surgery. Key exclusion criteria comprised active treatment with GLP‐1 analogues, pregnancy or breast‐feeding, cytostatic chemotherapy and/or radiation therapy within 6 months. For full in‐ and exclusion criteria of the GLORIOUS trial, see Table S1. Randomisation was performed via an internet‐based algorithm on the trial website, using permuted blocks of 4, 8 and 12. The two interventions were a priori assumed to be independent based on extensive literature review. An informed, written patient consent was obtained prior to study enrolment.

### 2.2 Substudy Specifications

This substudy was predefined by name and objective in the published GLORIOUS trial protocol [17]. However, as a detailed analysis plan was not prepublished, analyses should be regarded as post hoc exploratory and findings hypothesis‐generating.

The GLORIOUS trial included 1400 patients. The present substudy was designed for 800 patients. This sample size was determined by available resources at the time of GLORIOUS study launch. Enrolment in this substudy was voluntary, and patients were automatically offered enrolment if accepting GLORIOUS participation. Inclusion in this substudy began at GLORIOUS study launch and was consecutive. The GLORIOUS trial design and main results have been published previously [17, 18, 19]. No statistically significant interaction between the FiO_2_ and GLP‐1 interventions was found in the main GLORIOUS trial [18] nor in our substudy (all *p* for interaction > 0.2); therefore, the GLP‐1 intervention was considered independent.

### 2.3 GLP‐1 Intervention

The present study utilises the GLP‐1 intervention of the GLORIOUS trial. The study drug for this intervention was prepared as 1.5 mL of 20% human albumin to 248.5 mL of isotonic NaCl (placebo group). To this, 25 μg of exenatide (Byetta, Lilly) was added (GLP‐1 group). The preparation was performed by a trained nurse, who was unblinded to allocation, in a separate room and subsequently brought to the operating theatre. Infusion was initiated within 1 h of surgery start, and no later than at anaesthesia induction. Infusion was given via a central or peripheral intravenous line as a continuous infusion at a rate of 72 mL/h (0.12 μg/min.) for the first 15 min, followed by a lower rate of 26 mL/h (0.043 μg/min) for the next 6 h. Thus, a total of 17.4 μg of exenatide was administered to patients in the active group. Blood glucose levels were monitored closely during infusion. Any corrective glucose administration was initiated by the treating physician and documented. Patients deviating from protocol remained in the allocated group for data analysis. Allocation was blinded to patients, personnel in the operating room, investigators, and outcome assessors. The patients were not ventilated during CPB. Patients were kept normothermic (37°C) throughout the procedure and received standard peri‐ and post‐operative care, Table S2. The GLORIOUS trial was neutral in terms of GLP‐1 intervention, showing no significant difference in risk reduction between GLP‐1RA and placebo for the primary composite endpoint of death, renal failure, stroke or worsening heart failure [18].

### 2.4 Pulmonary Function Testing

Pulmonary function testing was performed before surgery at the pre‐operative visit, and post‐surgery at a 3‐month follow‐up visit.

This substudy was initially designed to include an additional pulmonary function test before discharge. However, this was abandoned after study launch due to organisational constraints, and because a larger than anticipated proportion of patients were unable to cooperate with spirometry testing at this point in their clinical course, reducing data collection below a meaningful level.

Pulmonary function testing comprised estimation of the DLCO as well as spirometry (FEV_1_, FVC, FEV_1_/FVC) utilising the NDD EasyOne Pro lung function testing device (NDD Medizintechnik AG Technoparkstrasse 1, CH‐8005 Zurich, Switzerland https://nddmed.com). Testing was performed by trained study personnel. Spirometry testing was performed according to established guidelines, reporting the best result of three good quality tests as evaluated by flow curves [20]. Spirometry measurements in this study included FEV_1_, and FVC in litres as well as FEV_1_ (% predicted) and FVC (% predicted), corresponding to the measured value compared to expected value, according to the Global Lung Function Initiative (GLI‐2012) reference values on age, sex, height, and ethnicity [21]. The FEV_1_/FVC presented are based on the ratio between unadjusted measures in crude litres. DLCO was measured by a single‐breath standard dilution gas method (10% helium, 0.3% carbon monoxide and 18%–25% oxygen) with a unit of mmol/min/kPa. DLCO (% predicted corrected) equals the measured DLCO (corrected for age, sex, hemoglobin and barometric pressure) compared to the expected DLCO according to the Global Lung Function Initiative (GLI‐2017) reference values by age, sex, height and ethnicity [22].

### 2.5 Endpoints

The co‐primary, exploratory endpoints were the difference between preoperative and three‐month postoperative DLCO (% predicted corrected) and FEV_1_/FVC. Secondary endpoints comprised the difference in FEV_1_ (% predicted) and FVC (% predicted). The % predicted values were chosen over crude values when available, to account for age, sex, height, and ethnicity.

### 2.6 Statistical Analysis

Descriptive statistics are presented as median with interquartile range (IQR) or mean with standard deviation (SD) as appropriate. Categorical variables are summarized using count and percentages. Postoperative change in pulmonary function compared to preoperative values—irrespective of treatment group—was analysed using paired *t*‐tests for normally distributed data or Wilcoxon signed‐rank test otherwise. Between‐group differences in postoperative pulmonary function change were analysed using mixed‐effects models. This approach was chosen to account for within‐subject variability over time and to handle missing data via a restricted maximum likelihood (REML) estimation. The missing data was assumed to be missing at random. The model included fixed effects for treatment group (GLP‐1RA receptor agonist or placebo), time (preoperative vs. postoperative) and their interaction term. The interaction term represents pulmonary function change by intervention group. As a random effect, patient‐ID was included to account for within‐subject variation. Reported output was the β‐coefficient with 95% confidence interval (CI) and *p*‐value for the interaction term, addressing magnitude, direction and statistical significance of treatment effect between groups. No imputation of missing values was performed. The study population was defined as all patients presenting a preoperative FEV_1_.

A subgroup analysis was conducted to investigate potential heterogeneity of treatment effect across age, sex, pre‐existing chronic pulmonary disease, duration of CPB and duration of reperfusion. Duration of reperfusion was defined as the time from aorta un‐clamping to removal of the aortic cannula. Continuous variables were dichotomized at their median for subgroup classification. Odds ratios for improvement or no change (favourable outcome) in pulmonary function at 3 months postoperative compared to preoperative values in the exenatide versus placebo group were calculated and presented using forest plots.

All analyses were performed in the intention‐to‐treat population. A two‐sided *p* value below 0.05 was considered statistically significant. *p* values were not adjusted for multiple testing according to the explorative nature of the study. Data management and statistical computation were performed in SAS Enterprise Guide 8.3 (Copyright SAS Institute Inc., Cary, NC, USA). Graphics were generated in RStudio (R Core Team 2021).

## 3 Results

A total of 882 GLORIOUS patients presented a preoperative FEV_1_ and were thus included in the study population. Four patients withdrew consent. The final study population comprised 878 patients. Of these, 444 patients were randomly allocated to the exenatide arm and 434 to the placebo arm, Figure S1. The two groups were balanced in baseline characteristics, Table 1 and procedure profiles, Table 2. The median age was 67.5 (IQR 60–74) years, with 151 (17%) of patients being female. A total of 94 patients (11%) presented with pre‐existing pulmonary disease, defined as known chronic obstructive pulmonary disease (COPD), asthma, or regular use of bronchodilators. The median EuroSCOREII was 1.4% (IQR 1.0–2.3). The majority of patients underwent isolated CABG (64%), while 220 (26%) underwent isolated SAVR and 87 (10%) a combination of both procedures.

**TABLE 1:** Baseline characteristics of the study population stratified by treatment group.

		Exenatide *n* = 444	Placebo *n* = 434
Age, years	Median (IQR)	68 (60, 74)	67 (60, 73)
Female sex	No. (%)	75 (17)	76 (18)
Caucasian race	No. (%)	431 (98)	429 (99)
Body mass index, kg/m^2^	Median (IQR)	27 (25, 30)	27 (25, 30)
Comorbidities			
Smoking, current or prior	No. (%)	295 (67)	278 (64)
Consumes alcohol	No. (%)	3 (0.7)	5 (1.2)
Heart failure	No. (%)	79 (18)	74 (17)
Hypertension	No. (%)	278 (62.9)	289 (66.9)
Type 2 diabetes	No. (%)	71 (16)	65 (15)
Type 1 diabetes	No. (%)	4 (0.9)	9 (2.1)
Pulmonary disease*	No. (%)	50 (11)	44 (10)
Ischemic heart disease	No. (%)	334 (76)	324 (75)
Previous myocardial infarction	No. (%)	78 (17.6)	81 (18.7)
Previous percutaneous coronary int.	No. (%)	67 (15.2)	57 (13.2)
Previous coronary artery bypass graft	No. (%)	4 (0.9)	2 (0.5)
Previous heart valve surgery	No. (%)	3 (0.7)	12 (2.8)
Previous stroke	No. (%)	42 (9.5)	32 (7.4)
Pacemaker or ICD	No. (%)	8 (1.8)	14 (3.2)
Dialysis	No. (%)	10 (2.3)	7 (1.6)
EuroSCOREII, %	Median (IQR)	1.5 (1.1, 2.3)	1.4 (1.0, 2.2)
NT‐proBNP, pmol/L	Median (IQR)	42 (16, 109)	35 (14, 100)
Creatinine, μmol/L	Median (IQR)	84 (75, 98)	85 (76, 101)
Cardiopulmonary performance			
Frailty score ≥ 4	No. (%)	138 (33)	145 (36)
NYHA class III‐IV	No. (%)	114 (27)	106 (26)
CCS class ≥ 3	No. (%)	54 (13)	52 (13)
LVEF, %	Median (IQR)	55 (45, 60)	55 (45, 60)
FEV_1_, L	Median (IQR)	2.6 (2.1, 3.1)	2.6 (2.1, 3.1)
FEV_1_ (% predicted)	Median (IQR)	87 (75, 98)	86 (76, 96)
FVC, L	Median (IQR)	3.5 (2.9, 4.1)	3.5 (2.9, 4.1)
FVC (% predicted)	Median (IQR)	89 (79, 101)	90 (79, 100)
FEV_1_ /FVC	Median (IQR)	0.76 (0.69, 0.80)	0.74 (0.70, 0.80)
DLCO (corrected), mmol/min/kPa	Median (IQR)	6.9 (5.4, 8.3)	6.7 (5.3, 8.1)
DLCO (% predicted corrected)	Median (IQR)	81 (70, 91)	79 (68, 89)
Medications			
Acetylsalicylic acid	No. (%)	271 (61.5)	266 (61.4)
Nonvitamin K oral anticoagulants	No. (%)	27 (6.1)	37 (8.5)
Angiotensin‐converting enzyme inhibitor	No. (%)	130 (29.5)	106 (24.5)
Angiotensin receptor blocker	No. (%)	105 (23.8)	122 (28.2)
Statins	No. (%)	308 (69.8)	313 (72.3)
β‐Blockers	No. (%)	189 (42.9)	204 (47.1)
Thiazides	No. (%)	70 (15.9)	76 (17.6)
Insulin	No. (%)	25 (5.7)	23 (5.3)

* Known COPD/asthma or regular use of broncohdilators; ICD, implantable cardioverter‐defibrillator; EuroSCORE II, European System for Cardiac Operative Risk Evaluation II; NT‐proBNP, N‐terminal pro‐B‐type natriuretic peptide; NYHA, New York Heart Association; CCS, Canadian Cardiovascular Society; LVEF, left ventricular ejection fraction; FEV_1_, forced expiratory volume in 1 s; FVC, forced vital capacity; DLCO, diffusing capacity of the lung for carbon monoxide.

**TABLE 2:** Procedural characteristics of the study population stratified by treatment group.

		Exenatide *n* = 444	Placebo *n* = 434
Primary indication for surgery			
CABG	No. (%)	282 (63.8)	278 (64.2)
SAVR	No. (%)	110 (24.9)	114 (26.3)
CABG + SAVR	No. (%)	50 (11.3)	41 (9.5)
Performed surgery			
CABG	No. (%)	286 (65)	277 (65)
SAVR	No. (%)	110 (25)	110 (26)
CABG + SAVR	No. (%)	46 (10)	41 (10)
Additional surgery			
Ascending aorta	No. (%)	4 (0.9)	7 (1.6)
Mitral valve	No. (%)	3 (0.7)	4 (0.9)
Tricuspid valve	No. (%)	1 (0.2)	0 (0.0)
Patent foramen ovale closure	No. (%)	0	0
Left atrial appendix closure	No. (%)	0	0
Duration of			
Cardiopulmonary bypass (CPB), min	Median (IQR)	92 (72, 120)	87 (69, 113)
Aortic cross clamp, min	Median (IQR)	58 (42, 84)	54 (40, 80)
Reperfusion, min	Median (IQR)	23 (17, 31)	22 (17, 29)

Abbreviations: CABG, coronary artery bypass grafting; SAVR, surgical aortic valve replacement.

### 3.1 Plasma Glucose Before and After Intervention

Plasma glucose levels before study drug infusion were similar in the exenatide and placebo groups (median 5.9 mmol/L [IQR 5.5–6.5] vs. 5.9 mmol/L [IQR 5.5–6.7], respectively). Immediate post‐intervention, the exenatide group showed significantly lower plasma glucose levels (median 5.3 mmol/L [IQR 4.7–6.4]) compared to placebo (median 6.7 mmol/L [IQR 5.9–7.8]); *p* < 0.005.

### 3.2 Differences in Pulmonary Function Between Preoperative and 3 Months Postoperative

At 3 months postoperative, pulmonary function declined significantly across all parameters, irrespective of intervention group, in the whole study cohort.

DLCO (% predicted corrected) declined from a preoperative median of 80% (IQR 68–90) to 72% (IQR 61–84), corresponding to a decline of 7.7 percentage points (pp) (95% CI 6.2 to 9.1); *p* < 0.001. The median FEV_1_/FVC ratio was 0.75 (IQR 0.69–0.80) preoperative, and 0.73 (IQR 0.68–0.78) postoperative, corresponding to a 1.6 (95% CI 1.0 to 2.1) decline; *p* < 0.001.

Correspondingly, FEV₁ (% predicted) declined from a preoperative median of 86% (IQR 75–97) to 81% (IQR 69–90) – a decline of 8.3 pp. (95% CI 6.6 to 8.3); *p* < 0.001, and FVC (% predicted) from 90% (IQR 79–99) to 85% (IQR 75–95), corresponding to a decline of 6.1 pp. (95% CI 5.2 to 7.0); *p* < 0.001.

### 3.3 Differences in Pulmonary Function Between Preoperative and 3 Months Postoperative by Intervention Group

**FIGURE 1:**
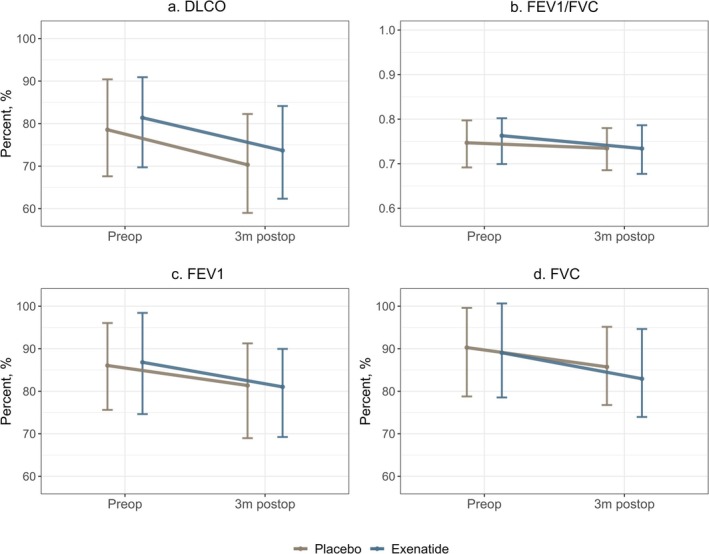
Pulmonary function preoperative and 3 months postoperative by intervention group Exenatide (blue); placebo (grey). Panel a: DLCO (% predicted corrected). Panel b: FEV_1_/FVC (the ratio of unadjusted, crude measures). Panel c: FEV_1_ (% predicted). Panel d: FVC (% predicted). Medians with IQR are shown. Sample sizes of the subgroups [preoperative; three‐months postoperative] in the placebo/exenatide group: Panel a: [224;141]/[241;152]; Panel b: [404;250]/[424;275]; Panel c: [434;305]/[444;330]; Panel d: [420;305]/[423;330].

The postoperative decline in pulmonary function did not differ between the exenatide and placebo groups. The estimated between‐group difference in postoperative decline was −0.2 pp. for DLCO (% predicted corrected) (95% CI −3.2 to 2.8; *p* for interaction = 0.9) and 0.001 for FEV₁/FVC (95% CI −0.001 to 0.01; *p* for interaction = 0.9), Table 3 and Figure 1.

**TABLE 3:** Pulmonary function preoperative and 3 months postoperative by treatment group for the primary endpoints. Pre‐ and postoperative values are presented as medians (IQR). The between‐group difference in change is presented as the beta‐coefficient (95% CI) from the mixed effects model, depicting the difference in decline between the two groups, including *p* for interaction. The FEV1/FVC is based on the ratio of crude, unadjusted values in litres.

	Preoperative	3 months postoperative	Between group difference in change
DLCO (% predicted corrected)			
Exenatide	81 (70, 91)	74 (62, 84)	−0.2 (−3.2 to 2.8)
Placebo	79 (68, 90)	70 (59, 82)	*p*_*Interaction*_ *=* 0.9
FEV_1_/FVC			
Exenatide	0.76 (0.70, 0.80)	0.73 (0.68, 0.79)	0.001 (−0.001 to 0.01)
Placebo	0.74 (0.69, 0.80)	0.73 (0.69, 0.78)	*p*_*Interaction*_ *=* 0.9

For secondary endpoints, the estimated between‐group difference in postoperative change was 0.3 pp. for FEV₁ (% predicted) (95% CI −2.2 to 1.7; *p* for interaction = 0.5), and 0.8 pp. for FVC (% predicted) (95% CI −2.7 to 1.0; *p* for interaction = 0.4), Figure 1.

### 3.4 Subgroup Analysis: Postoperative Change in Pulmonary Function by Intervention Group

**FIGURE 2:**
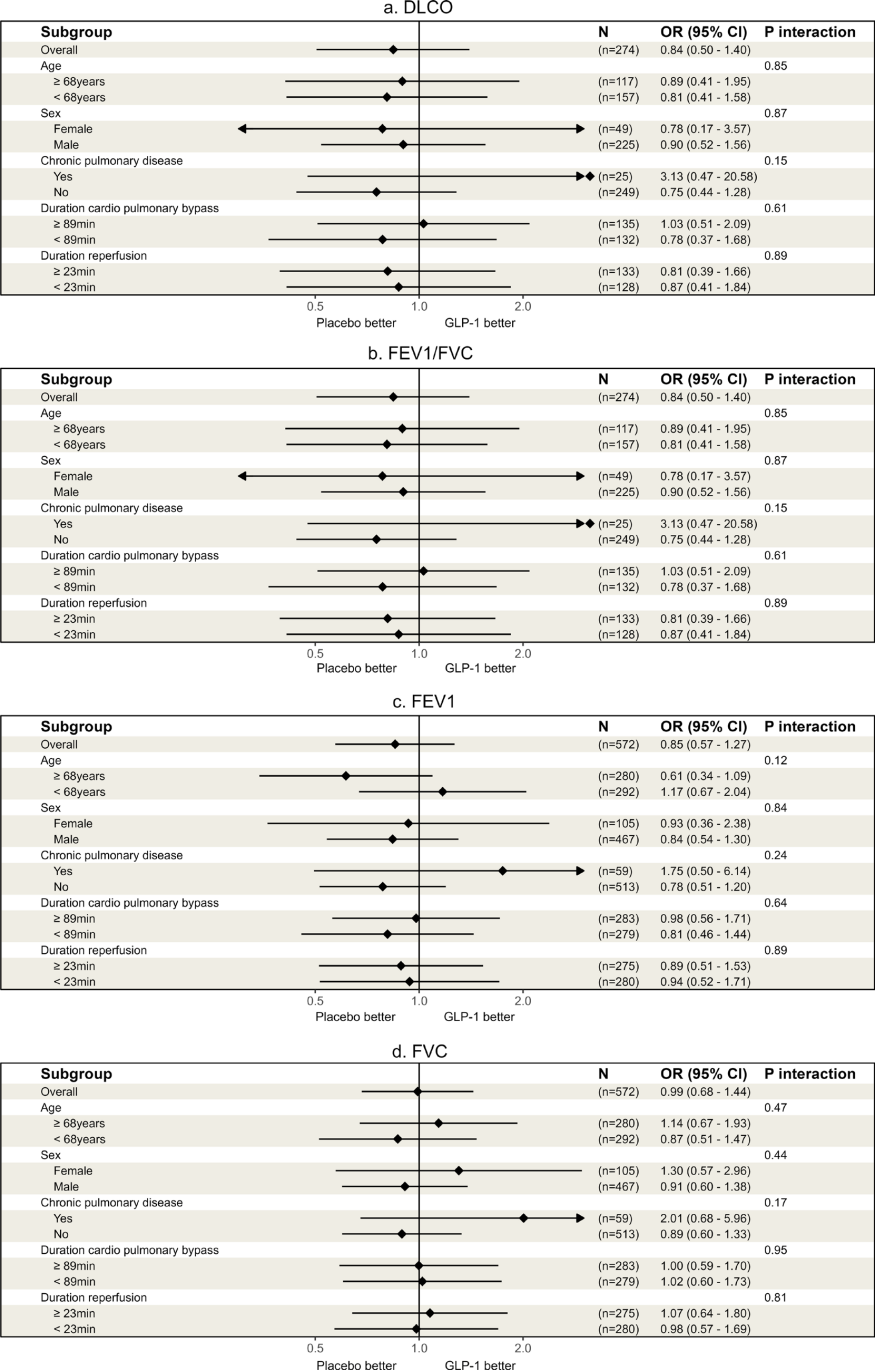
Subgroup analysis Forest Plot showing odds ratios (OR) for favourable outcome (improvement/no change in pulmonary function) at three months compared to preoperative values, in the GLP‐1RA vs. placebo group. Panel (a), FEV_1_ (% predicted); (b), FVC (% predicted); (c), FEV_1_/FVC (the ratio of unadjusted, crude measures); and (d), DLCO (% predicted corrected). N denotes the number of patients in each subgroup. Continuous subgroup variables were dichotomized at the median. An OR > 1 favours GLP‐1; OR < 1 favours placebo.

No differences in odds ratios for postoperative pulmonary function improvement or stability were observed across all specified subgroups, Figure 2. Additionally, no significant between‐group differences were found between each subgroup pair (data not shown).

## 4 Discussion

In this exploratory substudy of the GLORIOUS trial, including low‐risk patients undergoing non‐emergent, CPB‐assisted open‐heart surgery, we found mild‐to‐moderate (~6%–8%) reductions in both ventilatory properties and pulmonary diffusing capacity 3 months after index surgery. However, the GLP‐1RA exenatide did not mitigate this decline compared to placebo. Findings were consistent across subgroup analyses.

Our findings of a mild‐to‐moderate reduction in pulmonary function 3 months after surgery is consistent with the sparse existing evidence on longer‐term outcomes in this patient population [1, 2, 3, 23]. In a small observational cohort study including 25 adult patients, Westerdahl et al. [2] reported a 10% decrease in DLCO, and 13% reduction in FEV_1_ 4 months after CABG. While pulmonary dysfunction in the immediate postoperative phase of open‐heart surgery has received some research attention [4, 24], our data contribute to the limited evidence base on contemporary, longer‐term outcomes [1, 2, 3, 23].

The clinical impact of this pulmonary decline remains paramount, yet difficult to translate. We do not have data on patient‐centred outcomes such as New York Heart Association (NYHA) class. Regardless, previous studies have reported substantial discrepancies between subjective dyspnea and objective pulmonary function changes, including in this patient cohort [25]. It must be speculated that the symptom burden accompanying the observed pulmonary decline is likely modest in this relatively (pulmonary) healthy patient population. However, for patients with significant pre‐existing pulmonary disease, a 6%–8% decline in ventilatory function and diffusing capacity may prove more debilitating, which is why our findings cannot be extrapolated to such vulnerable patient subgroups.

Our neutral findings raise the question of whether the applied dose and timing of exenatide adequately support the study objective. The infusion regimen followed the protocol of Lønborg et al. [26], whose randomised trial demonstrated reduced myocardial infarct size in selected STEMI patients, proposedly through mitigation of myocardial reperfusion injury. Timing of intervention is likely pivotal, given the short half‐life of exenatide (approximately 2.4 h) [27]. In the present study, infusion comprised the entire length of CPB, including the critical pulmonary reperfusion phase during weaning, and extended into the early postoperative phase for most patients, lasting for a total of 6 h.

This study utilised the first‐generation GLP‐1RA, exenatide that was the subject of scientific interest during the GLORIOUS trial design. Newer, more potent GLP‐1RA like semaglutide, with a longer dose regimen, may prove beneficial in CPB‐related pulmonary protection in vulnerable patient subgroups. However, benefits are likely limited by the multifactorial etiology of pulmonary dysfunction after open‐heart surgery. In addition to inflammation‐ and ischaemia‐reperfusion injury, factors such as diaphragm dysfunction, altered chest wall mechanics, respiratory muscle weakness, and post sternotomy pain likely contribute significantly to the observed impairments [6, 28, 29]. Newer minimally invasive, off‐pump surgical techniques likely offer greater promise in postoperative pulmonary preservation. Future studies should employ even longer follow‐up (e.g., 1 year) and patient‐centred endpoints, to better capture clinically meaningful outcomes.

Our study has limitations. First, the GLORIOUS trial was conducted at a single, high‐volume, tertiary centre which may limit generalizability. Second, DLCO data exhibited substantial missingness (approximately 40% of patients lacked complete‐case data) due to prolonged periods of test gas shortages affecting DLCO measurement but not spirometry‐based variables. While no clear pattern in missingness was detected, this warrants cautious interpretation of results. Third, due to the exploratory nature of our study, no multiplicity adjustment was applied for the two primary endpoints. Lastly, our findings are limited to the three‐month scope. As postoperative sequelae are likely dynamic, longer‐term outcomes remain unknown.

In conclusion, pulmonary function in terms of ventilatory performance and diffusing capacity was mild‐to‐moderately reduced 3 months after CPB‐assisted heart surgery in this exploratory substudy of the GLORIOUS randomised clinical trial. However, an infusion of the GLP‐1RA exenatide did not mitigate this decline compared to placebo.

## Author Contributions

**Astrid Duus Mikkelsen:** data curation, formal analysis, investigation, methodology, project administration, resources, software, visualization, and writing of the original manuscript. **Christian Hassager:** conceptualization, validation, methodology, review and editing of the manuscript. **Jesper Kjærgaard** and **Sebastian Wiberg:** conceptualization, validation, supervision, data curation, methodology, review and editing of the manuscript. **Lars Køber:** conceptualization, validation, methodology, resources. **Peter Hasse Møller‐Sørensen**, **Dan Høfsten**, **Jens Christian Nilsson**, **Christian Holdflod Møller**, and **Hans Henrik Lawaetz Schultz:** conceptualization, data curation, validation, review and editing of the final manuscript.

## Funding

This study was supported by The Beckett Foundation and The Copenhagen University Hospital, Rigshospitalet's Research Foundation.

## Conflicts of Interest

A. D. M.: Financial support for salary was provided by The Beckett Foundation and The Copenhagen University Hospital, Rigshospitalet's Research Foundation. L. K.: Reports speaker's fee from Astra Zeneca, Boehringer, Novartis, and Novo. J. K.: Reports a research grant (NNF22OC0079649) outside the submitted work. The other authors declare no conflicts of interest.

## Supporting information


**Table S1:** Complete in‐ and exclusion criteria of the GLORIOUS trial. No additional in‐ or exclusion criteria applied for the present substudy. Participation in the substudy was a voluntary add‐on to participation in the parent GLORIOUS trial.
**Table S2:** Standard postoperative ICU treatment targets at the GLORIOUS trial study site for patients undergoing non‐emergent CABG and SAVR.
**Figure S1:** CONSORT diagram of patient flow. No additional in‐ or exclusion criteria applied for the present substudy. Participation in this substudy was a voluntary add‐on to participation in the parent GLORIOUS trial. Enrollment began at GLORIOUS study launch.

## Data Availability

The data that support the findings of this study are available from the corresponding author upon reasonable request.

## References

[1] E. Westerdahl , M. Jonsson , and M. Emtner , “Pulmonary Function and Health‐Related Quality of Life 1‐Year Follow Up After Cardiac Surgery,” Journal of Cardiothoracic Surgery 11 (2016): 99. 27390849 10.1186/s13019-016-0491-2 PMC4938995

[2] E. Westerdahl , B. Lindmark , I. Bryngelsson , and A. Tenling , “Pulmonary Function 4 Months After Coronary Artery Bypass Graft Surgery,” Respiratory Medicine 97 (2003): 317–322. 12693792 10.1053/rmed.2002.1424

[3] Z. Shenkman , Y. Shir , Y. G. Weiss , B. Bleiberg , and D. Gross , “The Effects of Cardiac Surgery on Early and Late Pulmonary Functions,” Acta Anaesthesiologica Scandinavica 41 (1997): 1193–1199. 9366943 10.1111/j.1399-6576.1997.tb04865.x

[4] T. G. Tanner and M. O. Colvin , “Pulmonary Complications of Cardiac Surgery,” Lung 198 (2020): 889–896. 33175990 10.1007/s00408-020-00405-7 PMC7655908

[5] D. Paparella , “Cardiopulmonary Bypass Induced Inflammation: Pathophysiology and Treatment. An Update,” European Journal of Cardio‐Thoracic Surgery 21 (2002): 232–244. 11825729 10.1016/s1010-7940(01)01099-5

[6] J. L. Huffmyer and D. S. Groves , “Pulmonary Complications of Cardiopulmonary Bypass,” Best Practice & Research. Clinical Anaesthesiology 29 (2015): 163–175. 26060028 10.1016/j.bpa.2015.04.002 PMC10068650

[7] X.‐M. Zheng , Z. Yang , G. L. Yang , Y. Huang , J. R. Peng , and M. J. Wu , “Lung Injury After Cardiopulmonary Bypass: Alternative Treatment Prospects,” World Journal of Clinical Cases 10 (2022): 753–761. 35127892 10.12998/wjcc.v10.i3.753 PMC8790450

[8] K. B. Buggeskov , R. G. Maltesen , B. S. Rasmussen , et al., “Lung Protection Strategies During Cardiopulmonary Bypass Affect the Composition of Blood Electrolytes and Metabolites—A Randomized Controlled Trial,” Journal of Clinical Medicine 7 (2018): 462. 30469433 10.3390/jcm7110462 PMC6262287

[9] J. Lønborg , N. Vejlstrup , H. Kelbæk , et al., “Exenatide Reduces Reperfusion Injury in Patients With ST‐Segment Elevation Myocardial Infarction,” European Heart Journal 33 (2012): 1491–1499. 21920963 10.1093/eurheartj/ehr309

[10] L. A. Nikolaidis , S. Mankad , G. G. Sokos , et al., “Effects of Glucagon‐Like Peptide‐1 in Patients With Acute Myocardial Infarction and Left Ventricular Dysfunction After Successful Reperfusion,” Circulation 109 (2004): 962–965. 14981009 10.1161/01.CIR.0000120505.91348.58

[11] J. J. Holst , “The Physiology of Glucagon‐Like Peptide 1,” Physiological Reviews 87 (2007): 1409–1439. 17928588 10.1152/physrev.00034.2006

[12] G. Muscogiuri , A. Cignarelli , F. Giorgino , et al., “GLP‐1: Benefits Beyond Pancreas,” Journal of Endocrinological Investigation 37 (2014): 1143–1153. 25107343 10.1007/s40618-014-0137-y

[13] J. Fandiño , L. Toba , L. C. González‐Matías , Y. Diz‐Chaves , and F. Mallo , “GLP‐1 receptor agonist ameliorates experimental lung fibrosis,” Scientific Reports 10 (2020): 18091. 33093510 10.1038/s41598-020-74912-1 PMC7581713

[14] W. Zhou , W. Shao , Y. Zhang , D. Liu , M. Liu , and T. Jin , “Glucagon‐Like Peptide‐1 Receptor Mediates the Beneficial Effect of Liraglutide in an Acute Lung Injury Mouse Model Involving the Thioredoxin‐Interacting Protein,” American Journal of Physiology‐Endocrinology and Metabolism 319 (2020): E568–E578. 32723174 10.1152/ajpendo.00292.2020 PMC7839242

[15] A. D. Altintas Dogan , O. Hilberg , S. Hess , et al., “Respiratory Effects of Treatment With a Glucagon‐Like Peptide‐1 Receptor Agonist in Patients Suffering From Obesity and Chronic Obstructive Pulmonary Disease,” International Journal of Chronic Obstructive Pulmonary Disease 17 (2022): 405–414. 35237033 10.2147/COPD.S350133 PMC8882670

[16] P. Rogliani , M. G. Matera , L. Calzetta , et al., “Long‐Term Observational Study on the Impact of GLP‐1R Agonists on Lung Function in Diabetic Patients,” Respiratory Medicine 154 (2019): 86–92. 31228775 10.1016/j.rmed.2019.06.015

[17] S. Wiberg , J. Kjaergaard , R. Møgelvang , et al., “Efficacy of a Glucagon‐Like Peptide‐1 Agonist and Restrictive Versus Liberal Oxygen Supply in Patients Undergoing Coronary Artery Bypass Grafting or Aortic Valve Replacement: Study Protocol for a 2‐By‐2 Factorial Designed, Randomised Clinical Trial,” BMJ Open 11 (2021): e052340. 10.1136/bmjopen-2021-052340 PMC8573662 34740932

[18] J. Kjaergaard , et al., “Efficacy of the Glucagon‐Like Peptide‐1 Agonist Exenatide in Patients Undergoing CABG or Aortic Valve Replacement: A Randomized Double‐Blind Clinical Trial,” Circulation. Cardiovascular Interventions 18 (2025): e014961, 10.1161/CIRCINTERVENTIONS.124.014961. 40265262

[19] S. Wiberg , et al., “Restrictive Versus Liberal Oxygenation in Patients Undergoing Cardiopulmonary Bypass‐Assisted Heart Surgery: A Randomised Controlled Trial,” British Journal of Anaesthesia 135 (2025): 1618–1625. S0007091225005331, 10.1016/j.bja.2025.08.005. 40975689 PMC12799424

[20] B. L. Graham , I. Steenbruggen , M. R. Miller , et al., “Standardization of Spirometry 2019 Update. An Official American Thoracic Society and European Respiratory Society Technical Statement,” American Journal of Respiratory and Critical Care Medicine 200 (2019): e70–e88. 31613151 10.1164/rccm.201908-1590ST PMC6794117

[21] P. H. Quanjer , S. Stanojevic , T. J. Cole , et al., “Multi‐Ethnic Reference Values for Spirometry for the 3–95‐Yr Age Range: The Global Lung Function 2012 Equations,” European Respiratory Journal 40 (2012): 1324–1343. 22743675 10.1183/09031936.00080312 PMC3786581

[22] S. Stanojevic , B. L. Graham , B. G. Cooper , et al., “Official ERS Technical Standards: Global Lung Function Initiative Reference Values for the Carbon Monoxide Transfer Factor for Caucasians,” European Respiratory Journal 50 (2017): 1700010. 28893868 10.1183/13993003.00010-2017

[23] H. Rouhi‐Boroujeni , H. Rouhi‐Boroujeni , P. Rouhi‐Boroujeni , and M. Sedehi , “Long‐Term Pulmonary Functional Status Following Coronary Artery Bypass Grafting Surgery,” ARYA Atherosclerosis 11 (2015): 163–166. 26405447 PMC4568203

[24] R. Badenes , A. Lozano , and F. J. Belda , “Postoperative Pulmonary Dysfunction and Mechanical Ventilation in Cardiac Surgery,” Critical Care Research and Practice (2015): 420513. 25705516 10.1155/2015/420513 PMC4332756

[25] M. K. Sahu , M. Yadav , M. P. Hote , S. P. Singh , and S. K. Choudhary , “Pulmonary Function Changes Before and After Mitral Valve Surgery in Severe Mitral Stenosis,” Journal of Cardiac Critical Care TSS 083, no. 02 (2018): 079.

[26] J. Lønborg , H. Kelbæk , N. Vejlstrup , et al., “Exenatide Reduces Final Infarct Size in Patients With ST‐Segment–Elevation Myocardial Infarction and Short‐Duration of Ischemia,” Circulation. Cardiovascular Interventions 5 (2012): 288–295. 22496084 10.1161/CIRCINTERVENTIONS.112.968388

[27] U.S. Food and Drug Administration , Exenatide (Byetta) Prescribing Information (2008), https://www.accessdata.fda.gov/drugsatfda_docs/label/2008/021773s012lbl.pdf.

[28] Á. Kristjánsdóttir , M. Ragnarsdóttir , P. Hannesson , H. J. Beck , and B. Torfason , “Respiratory Movements Are Altered Three Months and One Year Following Cardiac Surgery,” Scandinavian Cardiovascular Journal 38 (2004): 98–103. 15204235 10.1080/14017430410028492

[29] F. Maranta , L. Cianfanelli , V. Rizza , et al., “Diaphragm Dysfunction After Cardiac Surgery: Insights From Ultrasound Imaging During Cardiac Rehabilitation,” Ultrasound in Medicine & Biology 48 (2022): 1179–1189. 35351317 10.1016/j.ultrasmedbio.2022.02.011

