# Supplementary material for: Glucagon‐Like Peptide‐1 Agonist vs. Placebo and Pulmonary Decline After Open‐Heart Surgery: A Substudy of the GLORIOUS Randomised Clinical Trial
Source: Acta Anaesthesiol Scand. 2026 Jun 19;70(6):e70282. doi: 10.1111/aas.70282 (PMC13281132; doi:10.1111/aas.70282)

**SUPPLEMENT**

**Supplementary Table 1: Complete in- and exclusion criteria of the GLORIOUS trial.** No additional in- or exclusion criteria applied for the present substudy. Participation in the substudy was a voluntary add-on to participation in the parent GLORIOUS trial.

| Inclusion criteria |
| --- |
| 1. Appropriately obtained written informed consent. |
| 2. Age ≥18 years. |
| 3. Ischaemic heart disease requiring coronary artery bypass grafting and/or aortic valve disease requiring aortic valve replacement, irrespective of other concomitant valve surgery. |
| Exclusion criteria |
| 1. Active treatment with glucagon-like peptide-1 analogues. |
| 2. Obstructive hypertrophic cardiomyopathy, active myocarditis, constrictive pericarditis. |
| 3. Hyperthyroidism or untreated hypothyroidism. |
| 4. History of, or active pancreatitis. |
| 5. Acute surgery; subacute surgery (i.e. the following days) are eligible. |
| 6. Known allergy towards exenatide/Byetta or albumin (vehicle). |
| 7. On the urgent waiting list for a heart transplant (United Network of Organ Sharing category 1A or 1B or equivalent). |
| 8. Recipient of any major organ transplant. |
| 9. Receiving of has received cytotoxic or cytostatic chemotherapy and/ or radiation therapy for treatment of malignancy within 6 months before randomization. |
| 10. Clinical evidence of current malignancy, with the exceptions of: basal or squamous cell carcinoma, cervical intraepithelial neoplasia, prostate cancer with a life expectancy of >2.5. |
| 11. Currently enrolled in, or within 30 days from ending participation in other investigational drug trials for the treatment of diabetes or malignant obesity. Participation in other non-pharmacological trials is not an |

**Supplementary Table 2:** Standard postoperative ICU treatment targets at the GLORIOUS trial study site for patients undergoing non-emergent CABG and SAVR.

| Parameter | Target value |
| --- | --- |
| Middle arterial pressure (MAP) | 65-90 mmHg (8.7-12.0 kPa) |
| Central venous pressure (CVP) | <13 mmHg (<1.7 kPa) |
| Hemoglobin |  |
| - In case fully revascularized and preoperative LVEF > 30% - If signs of organ hypoperfusion | > 4.3 mmol/L  > 5.5 mmol/L |
| Diuresis | >1 ml/kg body weight/hour |
| Serum lactate | <2.0 mmol/L |
| Standard Base Excess (SBE) | -3 to +3 |
| Arterial oxygen saturation (SaO2) | > 94% |
| Arterial partial pressure of CO_2_ (PaCO_2_) | 37.5–45.0 mmHg *(5-6 kPa)* |
| Blood glucose | 6-10 mmol/L |
| Extubation within the first hour after ICU admission pursued. | |

**Supplementary Figure 1: CONSORT diagram of patient flow.** No additional in- or exclusion criteria applied for the present substudy. Participation in this substudy was a voluntary add-on to participation in the parent GLORIOUS trial. Enrollment began at GLORIOUS study launch.


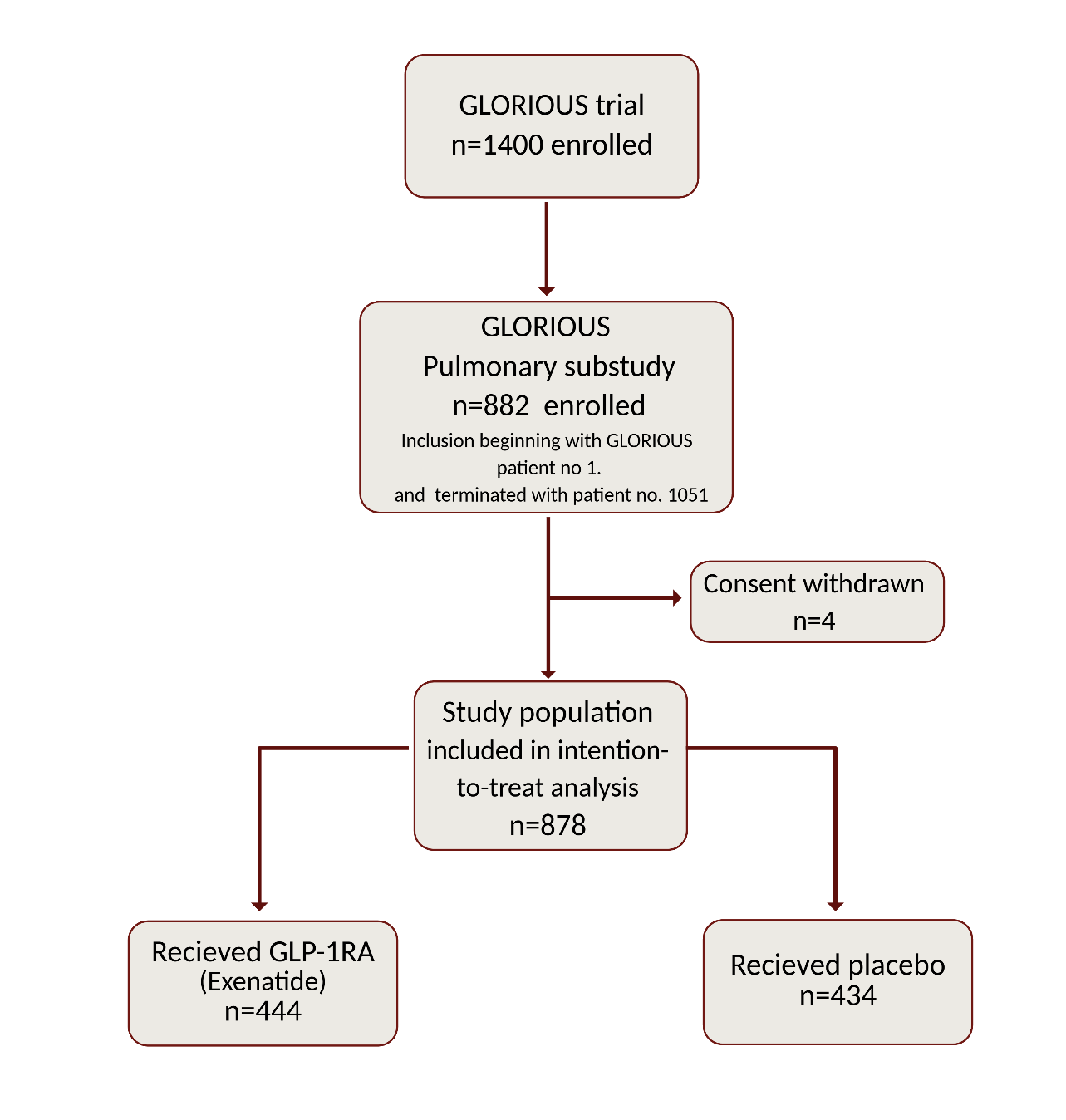

Supplement: Supplementary file 1 — Table S1: Complete in‐ and exclusion criteria of the GLORIOUS trial. No additional in‐ or exclusion criteria applied for the present substudy. Participation in the substudy was a voluntary add‐on to participation in the parent GLORIOUS trial. Table S2: Standard postoperative ICU treatment targets at the GLORIOUS trial study site for patients undergoing non‐emergent CABG and SAVR. Figure S1: CONSORT diagram of patient flow. No additional in‐ or exclusion criteria applied for the present substudy. Participation in this substudy was a voluntary add‐on to participation in the parent GLORIOUS trial. Enrollment began at GLORIOUS study launch. [file AAS-70-0-s001.docx]
